# Empowering Stroke Survivors: developing a patient version of guidelines to facilitate patient rehabilitation nursing of stroke patients with limb dysfunction in China

**DOI:** 10.3389/fpubh.2024.1482771

**Published:** 2025-01-07

**Authors:** Dan Yang, Jingyuan Zhang, Meiqi Meng, Xuejing Li, Lijiao Yan, Jiaxin Fang, Ziyan Wang, Sihan Chen, Xiaoyan Zhang, Yufang Hao, Fang Wang

**Affiliations:** ^1^School of Nursing, Beijing University of Chinese Medicine, Beijing, China; ^2^Institute of Basic Research in Clinical Medicine, China Academy of Chinese Medical Sciences, Beijing, China; ^3^Department of Vascular Surgery, Beijing Hospital, National Center of Gerontology, Institute of Geriatric Medicine, Chinese Academy of Medical Sciences, Beijing, China; ^4^Institute of Encephalopathy, Dongzhimen Hospital, Beijing University of Chinese Medicine, Beijing, China

**Keywords:** patient version of guidelines, rehabilitation care, stroke, evidence based nursing, limb dysfunction

## Abstract

**Objective:**

To develop a patient version of guidelines (PVG) for rehabilitation nursing (RN) in stroke patients with limb dysfunction, aiming to enhance patients’ awareness, self-management skills, and adherence to rehabilitation programs.

**Methods:**

This guideline was developed based on the cultural and healthcare context of China, and was guided on the Minimum standards for the Development Process, Content and Governance of Patient-Directed Knowledge Tools and the PVG tool book of the Guidelines International Network. The guideline was constructed through a normative process involving clarifying priority questions, assessing and integrating evidence, detailing and contextualizing recommendations, and evaluating the prototype of PVG.

**Results:**

Fifteen priority RN issues were identified, and eight articles (four guidelines and four evidence summaries) were included, all demonstrating robust methodological quality. The final guideline encompassed five themes: disease knowledge, functional assessment, symptom prevention and nursing, rehabilitation training, and traditional Chinese medicine nursing - a specialized approach integrating traditional Chinese medicine principles with modern nursing practices, including 26 recommendations.

**Conclusion:**

This patient-centered guideline, grounded in a robust scientific framework and tailored to patient needs, serves as a valuable reference for the RN of stroke patients with limb dysfunction. The development of context-specific patient guidelines that integrate best available evidence remains an area requiring continued effort and refinement. Further research is warranted to evaluate the implementation and effectiveness of this guideline within diverse Chinese healthcare context.

## 1 Introduction

Stroke, a prevalent acute cerebrovascular disorder, is the second leading cause of adult mortality globally and a primary contributor to long-term severe disability (1). Limb function impairment is a common sequela, with epidemiological studies showing high incidence rates. In the United States, over 80% of stroke patients experience lower limb dysfunction, with 25% retaining residual disabilities despite rehabilitation (2, 3). Similarly, in China, 85% of patients initially present with upper limb dysfunction, and 30–36% continue to exhibit impairments 6 months post-onset (4). Rehabilitation nursing (RN) has been empirically validated as an efficacious approach to mitigating disability rates in these patients (5), demonstrating potential in alleviating functional deficits, enhancing functional status, preventing complications, and improving daily living activities (6).

RN plays a crucial role in promoting limb function recovery in stroke patients through various interventions. In China, economic and geographical constraints commonly necessitate home-based rehabilitation (7, 8), potentially limiting patients’ access to professional, systematic therapy during the critical early rehabilitation phase (9). Despite stroke patients’ desire to learn rehabilitation skills, they often lack awareness of sustained care needs (10). While online health education has emerged as a solution, existing materials frequently lack scientific rigor or contain outdated information. There is an urgent need for evidence-based RN knowledge specific to stroke-induced limb function impairment to enhance patient and caregiver awareness and adherence.

Clinical practice guidelines (CPGs) are widely recognized as the most scientifically rigorous source of health recommendations (11). However, the professional terminology and complex medical knowledge they contain often render them difficult for patients and the general public to comprehend and apply. To address this issue, patient versions of guideline (PVG) have emerged as a solution. PVG (12) refer to documents that “translate” the recommendations and underlying principles of CPGs into a format more accessible and applicable for patients and the general public. The Guidelines International Network (GIN) published its first handbook for developing PVGs in 2015, with an updated version released in 2021 (13). In 2018, the National Institute of Health Care of Southern Netherlands (14) established the Minimum Criteria for the Development Process, Content and Governance of Patient-Directed Knowledge Tools [PDKT(MC-PCG)]. In 2021, GIN (15) formulated a reporting checklist for PVGs. These methodological resources provide researchers with guidance for developing PVGs.

Although there are currently no specific CPGs or PVGs for RN of stroke with limb dysfunction, relevant content has been addressed in existing guidelines. For instance, the American Heart Association/American Stroke Association (AHA/ASA) published “Guidelines for Adult Stroke Rehabilitation and Recovery: A Guideline for Healthcare Professionals From the American Heart Association/American Stroke Association” in 2016 (16), which outlines general principles and strategies for stroke rehabilitation. Similarly, the “Clinical Management Guidelines for Cerebrovascular Diseases” (17), released by the Chinese Stroke Association in 2019, emphasizes the importance of early and continuous rehabilitation. These guidelines provide substantial evidence-based support for the RN of patients with stroke-induced limb function impairment.

This study aims to develop a PVG specifically for the RN of stroke patients with limb dysfunction. Guided by international PVG development standards and considering the Chinese context, this research will construct a patient-centered, comprehensive, and practical PVG for the RN of stroke patients with limb dysfunction. This PVG will fill a critical gap in China, providing evidence-based guidance for limb RN in stroke patients, thereby enhancing rehabilitation outcomes and quality of life. Additionally, the PVG will serve as a reference for healthcare providers, facilitating patient-provider communication and improving RN services.

## 2 Methods

This study employs the PDKT(MC-PCG) and the GIN’s PVG toolkit as theoretical frameworks. The GIN toolkit outlines key elements in PVG development, including stakeholder engagement and content presentation. The PDKT(MC-PCG), as the first internationally applicable tool for PVG development, provides a systematic approach covering team formation, scope definition, patient needs identification, and content determination. As PDKT(MC-PCG) primarily targets patient versions of individual CPGs, and no specific CPG exists for RN of stroke patients with limb dysfunction, our team, based on a literature review (18–20) and expert consultations, opted to systematically search for evidence meeting identified patient needs. We will integrate the best evidence using guideline adaptation methods (21, 22), aiming to synthesize current best practices in RN for stroke-induced limb function impairment and translate them into a PVG.

**Figure 1:**
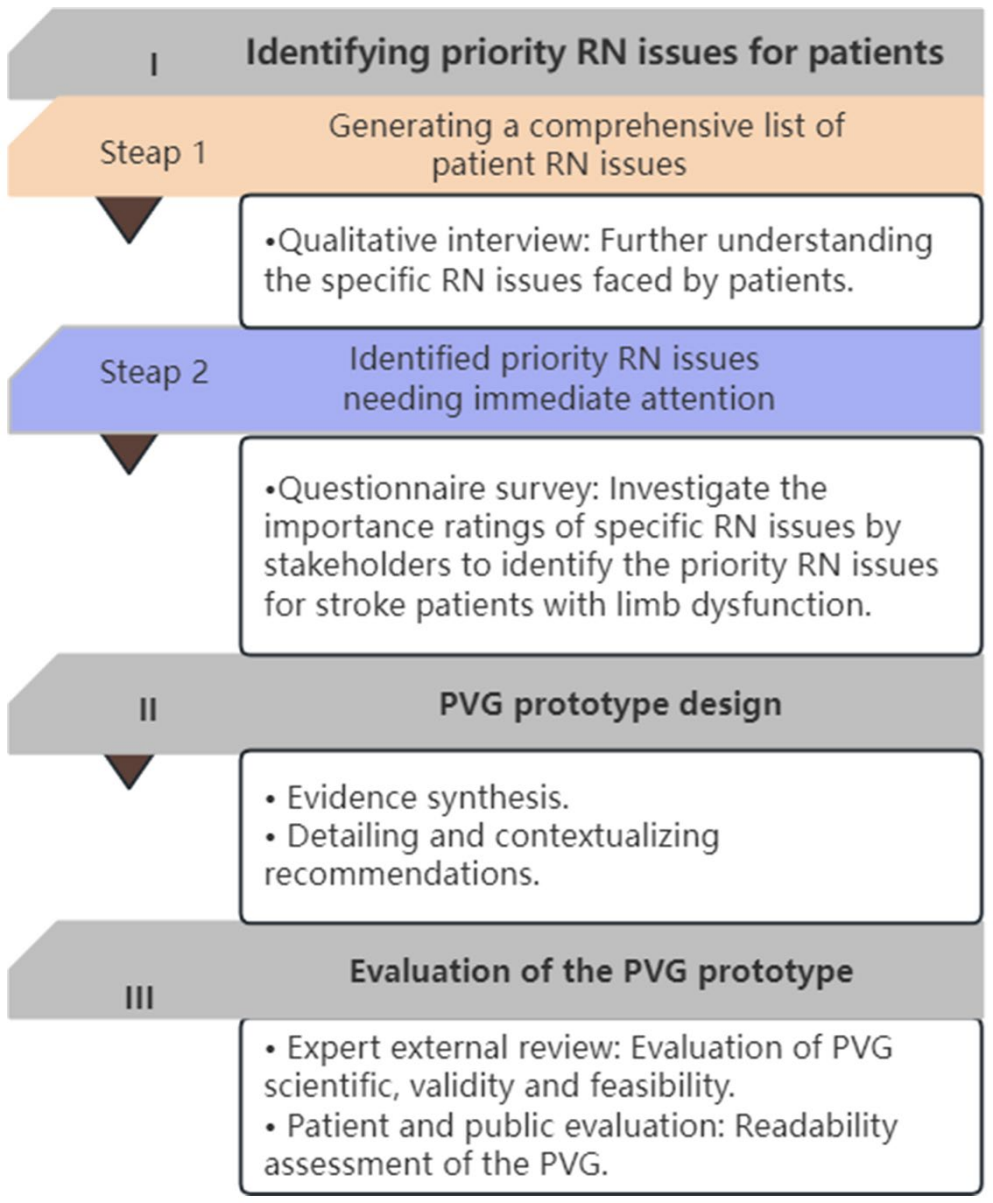
The PVG development flowchart based on PDKT (MC-PCG) and GIN’s PVG tools.

The study comprises three phases (see Figure 1): (1) Identifying priority RN issues for patients; (2) Developing a prototype of the PVG content based on the identified issues; and (3) evaluating the prototype of the PVG for readability, comprehensibility, and usability. This PVG has been registered on the International Practice Guidelines Registry Platform^1^ with the registration number IPGRP-2020CN203.

At the start, we established a 25 member guideline development team, comprising five functional groups: development, consensus, evidence evaluation and translation, patient and public, and external review. Team members included healthcare providers, research experts, patient representatives, and health education editors. Detailed information on group members and their responsibilities is available in Appendix A.

### 2.1 Identifying priority RN issues for patients

This phase began with semi-structured interviews to identify RN issues faced by patients, generating a comprehensive list. We then developed a questionnaire based on this list and surveyed patients and healthcare providers, asking them to rank the importance of each issue. This process identified priority RN issues needing immediate attention.

#### 2.1.1 Qualitative interview

**Figure 2:**
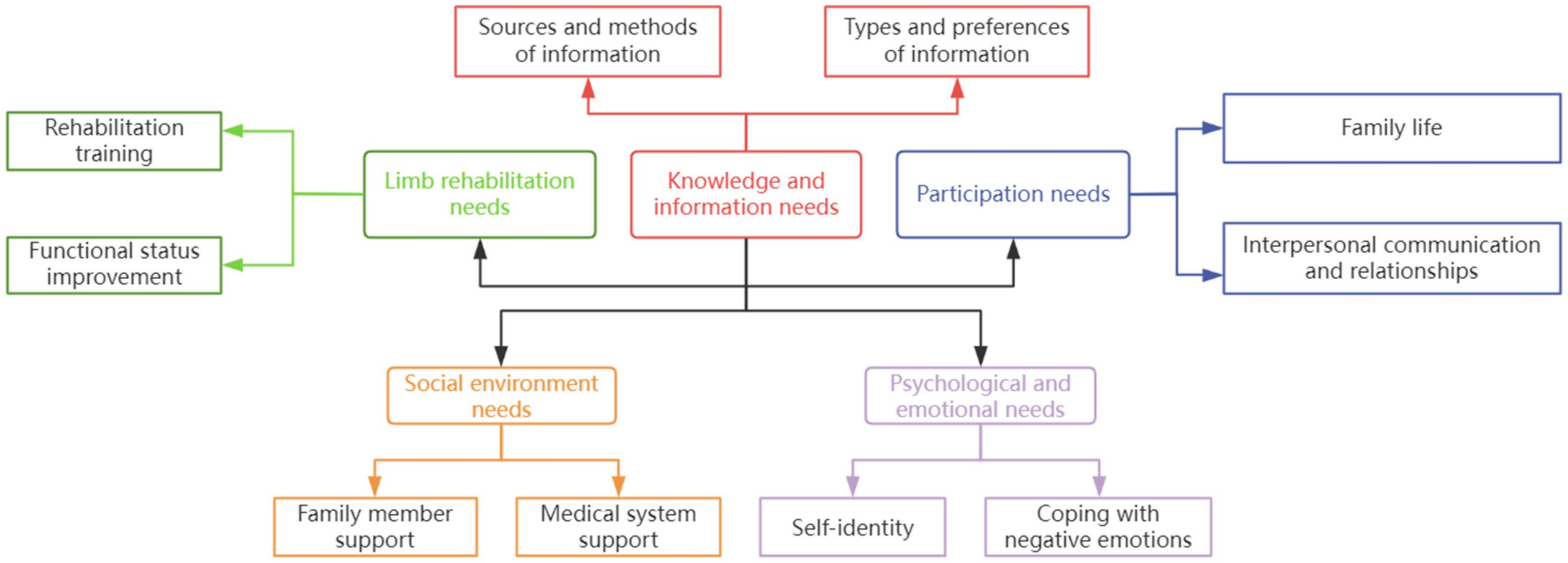
Thematic map of RN needs for stroke patients with limb dysfunction.

A semi-structured interview guide was developed based on RN themes from previous literature (23), comprising five primary and 10 secondary themes (Figure 2). Stroke patients with limb dysfunction (and their caregivers) and healthcare providers were purposively sampled from Beijing Dongzhimen Hospital’s Encephalopathy Department (August–September 2021). Each interview lasted approximately 40 min. The sample size for our interviews was determined by the principle of information saturation, whereby sampling was discontinued when no new codes emerged from the interview data (24). Deductive content analysis (25) was used, with an initial coding framework based on the International Classification of Functioning, Disability and Health (ICF). This process generated a comprehensive list of RN issues, described using evidence-based question formulation (26). The ICF (27, 28), widely used to classify functioning, disability, and health, facilitated a holistic analysis of patients’ rehabilitation needs, encompassing functional disabilities and contextual factors.

#### 2.1.2 Questionnaire survey

Using purposive sampling, stroke patients with limb function impairment, caregivers, and healthcare providers from Dongzhimen Hospital’s Encephalopathy Department were surveyed (October–November 2021). A self-designed questionnaire based on the RN issue list used a 5-point Likert scale (1 = not important, 5 = crucial). SPSS 26.0 was used for descriptive analysis, with 10–15 priority issues identified through expert consensus (29).

### 2.2 PVG prototype design

This phase involved designing the PVG prototype through targeted literature review and expert consultation. It comprised two main components: evidence synthesis, and the detailing and contextualization of recommendations.

#### 2.2.1 Evidence synthesis

##### 2.2.1.1 Evidence search and quality appraisal

A systematic search (30) based on identified RN issues was conducted across international guideline websites, Chinese and English databases, and stroke-specific professional association websites from January 2016 to December 2021 (search strategies and criteria in Appendix B). Two evidence-based medicine experts independently screened the literature, with disagreements resolved through discussion. Included literature was independently evaluated using appropriate quality assessment tools: AGREE II (31) for CPG and CASE worksheet (32) for evidence summaries.

##### 2.2.1.2 Evidence integration and analysis

Due to the diverse sources of eligible evidence, we established principles for evidence integration (33): (1) Select concise, clear evidence when consistent; (2) Combine complementary evidence logically; (3) Prioritize high - quality, latest authoritative literature when contradictory; (4) Retain original statements for independent evidence. The SIGN PVG (34) recommendation grading system was used for presentation. Two researchers integrated evidence, with five clinical experts evaluating synthesis results and applicability. Modifications were made based on feedback. The overall expert authority coefficient (sum of individual coefficients divided by number of experts) was assessed, with >0.7 indicating acceptable expert consensus.

#### 2.2.2 Detailing and contextualizing recommendations

The evidence detailing phase aimed to supplement recommendations while maintaining consistency with original guidelines. Following GIN’s PVG toolkit principles (20), two researchers refined content focusing on structure rationality, appropriateness, clinical significance, and accuracy. The evidence translation phase then aligned detailed content with patients’ needs and enhanced comprehensibility. Guided by the Patient Education Materials Assessment Tool (PEMAT) (35), we emphasized ‘understandability’ and ‘actionability’. Healthcare providers, patient representatives, and science communicators evaluated the results, with researchers incorporating feedback for improvements. (Detailed principles and methods in Appendix C). This process ensured scientifically robust recommendations tailored to patients’ practical needs and understanding levels.

### 2.3 Evaluation of the PVG prototype

An external review panel (peer experts, patient representatives, and health science communicators) assessed the PVG content and development process. Revisions were made based on their feedback.

#### 2.3.1 Evaluation of PVG scientific, validity. and feasibility

An expert panel of evidence-based methodology specialists and stroke experts evaluated the PVG using a custom-designed “Expert Consultation Form.” The evaluation covered: (1) Significance of identified RN questions. (2)Evidence synthesis and translation processes. (3)Guideline recommendations’ usability, using the JBI FAME framework (36) (rated as “Agree,” “Unclear,” or “Disagree”). And(4)PVG reporting quality, using the Reporting checklist for public versions of guidelines (RIGHT-PVG) (15) (four domains, 12 topics, 17 items; rated as “Fully met,” “Partially met,” or “Not met”).

#### 2.3.2 Readability assessment of the PVG

Patient representatives and health science communicators evaluated the guideline’s readability using the Suitability Assessment of Materials (SAM) scale. This scale, developed by Doak et al. (37) and translated into Chinese by Li (38), assesses the readability of health education materials. Notably, the SAM scale has also been applied to evaluate the readability of PVG in previous studies (39).

## 3 Results

### 3.1 Priority RN issues for patients

#### 3.1.1 Qualitative interviews

We interviewed nine stroke patients with limb functional impairment (six males, three females; mean age 58 ± 9.04 years) and six neurology specialist healthcare providers (mean specialized experience 13.33 ± 6.19 years). Combining interview findings with literature review results, we synthesized a refined categorization of rehabilitation nursing needs. This resulted in five primary themes, 10 secondary themes, and 26 specific rehabilitation nursing issues for stroke patients with limb functional impairment (detailed list in Appendix D).

#### 3.1.2 Questionnaire survey

A total of 140 distributed questionnaires among three groups: caregivers, patients, and healthcare providers. Of these, 134 valid responses were received, resulting in an overall response rate of 95.71%. The valid responses were distributed as follows: 58 from caregivers (43.28% of valid responses), 44 from patients (32.84% of valid responses), and 32 from healthcare providers (23.88% of valid responses). Based on these responses, the guideline development group identified the top 15 priority RN issues for stroke patients with limb dysfunction. Detailed scoring results are in Table 1.

**Table 1:** Scores of thematic priorities for rehabilitation nursing issues.

Ranking	Thematic priorities for RN issues	Score (x̄ ± s)
1	Knowledge of stroke prevention	4.26 ± 0.60
2	Timing of early rehabilitation nursing	4.25 ± 0.60
3	Prevention of falls	4.22 ± 0.61
4	Prevention of skin damage	4.18 ± 0.62
5	Prevention/relief of spasticity symptoms	4.11 ± 0.62
6	Proper limb positioning	4.05 ± 0.63
7	Standing training	4.00 ± 0.63
8	Limb function assessment	3.99 ± 0.62
9	Position transfer	3.89 ± 0.63
10	Joint mobility training	3.85 ± 0.62
11	Rehabilitation training time	3.80 ± 0.61
12	Prevention/relief of shoulder pain symptoms	3.71 ± 0.60
13	Psychological nursing	3.56 ± 0.58
14	Traditional Chinese medicine nursing	3.49 ± 0.55
15	Prevention of deep vein thrombosis	3.22 ± 0.79

### 3.2 PVG prototype

#### 3.2.1 Evidence synthesis

The initial literature search yielded 205 articles. After applying inclusion and exclusion criteria, four guidelines (16, 17, 40, 41) and 4 evidence summaries (42–45) were ultimately included (see Table 2). All selected materials demonstrated good quality of evidence and were incorporated following intra-group discussions. (The included evidence and evaluation results are detailed in Appendix E).

**Table 2:** Characteristics of the included literature.

Included literature	Publication year	Literature theme	Literature type
China stroke Association (17)	2019	Standing training, walking training, daily activity ability training	CPG
Chinese Society of Neurology, Chinese Medical Association (40)	2017	Early rehabilitation, good limb placement, standing training, walking training	CPG
National Health and Family Planning Commission Stroke Prevention and Control Committee (41)	2017/2021(Updated)	ADL assessment, upright sitting, muscle strength training	CPG
American Heart/Stroke Association (16)	2016	Rehabilitation management, long-term rehabilitation guidance	CPG
Shanxi Provincial Hospital of Traditional Chine (42)	2019	Upper and lower limb movement therapy, gait training, posture training	Summary evidence
Southern Theater Command General Hospital of the People’s Liberation Army (43)	2020	Proper positioning, standing training, complication prevention, and muscle training.	Summary evidence
Renmin Hospital of Wuhan University (44)	2020	Physical Function Exercise	Summary evidence
Beijing University of Chinese Medicine (45)	2019	Chinese traditional medical nursing	Summary evidence

##### 3.2.1.1 Results of recommendation extraction

Three specialists from the study’s expert consensus group conducted the consultation, with an overall expert authority coefficient of 0.90. From 83 initial recommendations, 10 were excluded: nine for being beyond patient implementation scope and one for irrelevance to post-stroke limb functional impairment rehabilitation (details in Table 3), which resulted in 73 preliminarily included recommendations.

**Table 3:** Delete recommendations and reasons.

Deletion reason	Deleted recommendations
This falls within the scope of a nurse/therapist’s responsibilities, as the patient is unable to operate (or has difficulty understanding).	1. All stroke patients should be assessed for the risk of deep venous thrombosis (DVT) in the lower extremities. Severe stroke, bed rest, immobilization, heart failure, infection, dehydration, limb fractures, and other factors are risk factors for DVT formation in the acute phase of stroke. Early mobilization and rehabilitation are effective methods for preventing DVT (40). 2. It is reasonable to assess the fall risk of stroke patients annually using an appropriate tool (40). 3. Conduct nursing assessments based on pressure injury risk scores and provide nursing interventions accordingly (41). 4. For post-stroke shoulder pain and shoulder care, especially before discharge or care transitions, patient and family education (i.e., range of motion, transitional positioning) is recommended (43). 5. Early rehabilitation, occupational therapy, constraint-induced movement therapy, virtual reality rehabilitation training, functional electrical stimulation, and repetitive transcranial magnetic stimulation are recommended to improve daily living abilities (17). 6. The use of structured depression scales, such as the Patient Health Questionnaire, is recommended for routine post-stroke depression screening, stroke education, and providing appropriate advice (17). 7. The Beck Anxiety Inventory is recommended for assessing post-stroke anxiety (17). 8. Motivational interviewing and personalized education are recommended to identify individual risk factors, which may be beneficial for the long-term control of stroke risk factors (17). 9. It is recommended to follow up with discharged patients (44).
Not related to the thematic priorities of rehabilitation nursing for post-stroke limb dysfunction.	1. Improve the living and home environment of stroke patients (40).

##### 3.2.1.2 Integration of recommendations

Through extraction, classification, synthesis, and expert consultation, a final set of 26 recommendations was formulated, comprising: one on functional assessment, 10 on symptom prevention and care, 10 on rehabilitation training series, three on traditional Chinese medicine nursing, and two on disease knowledge. Detailed information is provided in Appendix E.

#### 3.2.2 Detailing and contextualizing recommendations

Consultations were conducted with four experts (authority coefficient: 0.88), two patient representatives, and one health science communicator.

Experts validated the accuracy and clinical significance of the detailed recommendations. However, they advised removing “Chinese herbal fumigation” and “muscle strength assessment” as unsuitable for home use or self-assessment. For the remaining recommendations, experts suggested considering specific application scenarios and adjusting according to contextual environments during implementation. (Final detailed recommendations are presented in Appendix F).

Regarding the contextualization of the detailed recommendations, experts confirmed the accuracy of the adapted content. They suggested presenting training procedures predominantly through a combination of text, images/videos, and step-by-step explanations, and providing more specific descriptions of recommendation strengths. Patient representatives and the communicator, while noting good readability, suggested: (1) explaining stroke-related limb impairment causes, (2) highlighting key information visually, and (3) detailing recommendation strengths to indicate relative importance. These inputs informed the PVG prototype development (Appendix G).

### 3.3 Evaluation of the PVG prototype

#### 3.3.1 Expert evaluation results

Five experts in evidence-based medicine/nursing and clinical management/nursing evaluated the study (expert authority coefficient: 0.87).

##### 3.3.1.1 PVG priority issues assessment results

Experts rated all 15 RN issues as highly important (mean scores ≥4; details in Appendix H).

##### 3.3.1.2 Evaluation results of PVG recommendation integration, detailing and contextualizing recommendations

Experts highly agreed on the content accuracy and theme appropriateness of the synthesized, detailed, and contextualized recommendations. They emphasized the importance of professional guidance when implementing nursing procedures from this PVG (details in Table 4).

**Table 4:** External review results of the integration and translation of recommendations (*n* = 5).

Recommendation (Number)		Integrated translation			Content accuracy			Thematic appropriateness		
		Agree	Unclear	Disagree	Agree	Unclear	Disagree	Agree	Unclear	Disagree
1. Functional assessment	1	4	1	0	5	0	0	5	0	0
2. Prevention and care of risks and symptoms	2.1	5	0	0	5	0	0	5	0	0
	2.2	5	0	0	5	0	0	5	0	0
	2.3	5	0	0	5	0	0	5	0	0
	2.4	5	0	0	5	0	0	5	0	0
	2.5	5	0	0	5	0	0	5	0	0
3. Training and rehabilitation	3.1	5	0	0	5	0	0	5	0	0
	3.2	5	0	0	5	0	0	5	0	0
	3.3.1	5	0	0	5	0	0	5	0	0
	3.3.2	5	0	0	5	0	0	5	0	0
	3.3.3	5	0	0	5	0	0	5	0	0
	3.3.4	5	0	0	5	0	0	5	0	0
4. Traditional Chinese medicine nursing	4.1	4	1	0	5	0	0	5	0	0
	4.2	4	1	0	5	0	0	5	0	0
5. Disease knowledge	5.1	5	0	0	5	0	0	5	0	0
	5.2	5	0	0	5	0	0	5	0	0

##### 3.3.1.3 Applicability assessment results of recommendations

Experts highly agreed on the recommendations’ feasibility, appropriateness, clinical significance, and effectiveness, indicating their alignment with Chinese clinical practice and potential for widespread application (details in Table 5).

**Table 5:** External review results of the applicability of recommendations (*n* = 5).

Recommendation (Number)		Feasibility			Appropriateness			Clinical significance			Effectiveness		
		Agree	Unclear	Disagree	Agree	Unclear	Disagree	Agree	Unclear	Disagree	Agree	Unclear	Disagree
1. Functional assessment	1	4	1	0	5	0	0	5	0	0	5	0	0
2. Prevention and care of risks and symptoms	2.1	5	0	0	5	0	0	5	0	0	5	0	0
	2.2	5	0	0	5	0	0	5	0	0	5	0	0
	2.3	5	0	0	5	0	0	5	0	0	5	0	0
	2.4	5	0	0	5	0	0	5	0	0	5	0	0
	2.5	5	0	0	5	0	0	5	0	0	5	0	0
3. Training and rehabilitation	3.1	5	0	0	5	0	0	5	0	0	5	0	0
	3.2	5	0	0	5	0	0	5	0	0	5	0	0
	3.3.1	5	0	0	5	0	0	5	0	0	5	0	0
	3.3.2	5	0	0	5	0	0	5	0	0	5	0	0
	3.3.3	5	0	0	5	0	0	5	0	0	5	0	0
	3.3.4	5	0	0	5	0	0	5	0	0	5	0	0
4. Traditional Chinese medicine nursing	4.1	4	1	0	4	1	0	5	0	0	4	1	0
	4.2	4	1	0	4	0	1	5	0	0	4	0	1
5. Disease knowledge	5.1	5	0	0	5	0	0	5	0	0	5	0	0
	5.2	5	0	0	5	0	0	5	0	0	5	0	0

##### 3.3.1.4 Evaluation results of PVG Reporting

Experts highly agreed on most reporting standards of the RN PVG for stroke patients with limb functional impairment, but suggested refinements in terminology, abbreviations, and question framing (details in Table 6).

**Table 6:** Evaluation of the PVG report (*n* = 5).

Section	Topic	Number	Evaluation		
			Fully met	Partially met	Not met
Basic information	Title/cover/copyright	1a	5	0	0
		1b	5	0	0
		1c	5	0	0
	Contact information	2	5	0	0
	Summary	3	5	0	0
Background	Introduction of the target topic	4a	5	0	0
		4b	5	0	0
	Purpose, scope, and target users	5	5	0	0
	Link to the source guideline	6	5	0	0
Recommendations	Recommendations	7a	5	0	0
		7b	5	0	0
		7c	5	0	0
	The strength of the recommendations and certainty of the evidence	8	5	0	0
Other information	Questions to ask	9	3	2	0
	Terms and abbreviations	10	1	4	0
	Funding	11	5	0	0
	Conflicts of interest	12	4	1	0

#### 3.3.2 Readability evaluation results of PVG by patients and health science communicator

Two patients and one health science communicator evaluated the readability of the PVG. The overall readability score for the PVG textual materials was (36.33 ± 1.53), with individual overall score percentages of 79.54, 81.81, and 86.36%, respectively. All three reviewers rated the PVG as excellent (evaluation results are presented in Table 7).

**Table 7:** Readability score of PVG in RN for limb dysfunction after stroke.

Dimension (6)	Number of Items (22)	Score range	Score (x̄ ± s)
Material content	4	7 ~ 8	7.33 ± 0.57
Literacy needs	5	4 ~ 7	5.33 ± 1.75
Charts and diagrams	5	8 ~ 10	8.67 ± 1.15
Layout design	3	4 ~ 5	4.67 ± 0.58
Learning incentives and motivation	3	5 ~ 6	5.33 ± 0.58
Cultural appropriateness	2	3 ~ 4	3.67 ± 0.58
Readability	22	3 ~ 10	36.33 ± 1.53

#### 3.3.3 Improvement of PVG prototype

Incorporating expert, patient, and public feedback, the team revised the initial PVG draft, producing the ‘2022 Patient Guideline for RN of Stroke-Related Limb Functional Impairment’ with a WeChat version (Appendix I). Main contents are in Table 8.

**Table 8:** PVG content of RN for limb dysfunction after stroke.

Catalogue	Overview of the content
➢ Introduction to PVG	Description of PVG target audience Summary of PVG content Methodology for PVG development Supplementary information related to PVG
➢ Understanding limb dysfunction in stroke	Overview of stroke Causes of limb dysfunction in stroke patients Consequences of limb dysfunction Importance of RN in stroke recovery
➢ Knowledge of physical RN	Importance of functional assessment Fall prevention strategies Techniques for shoulder pain prevention Methods for spasticity prevention and relief Skin protection measures Strategies for deep vein thrombosis prevention
➢ Implementation of RN	Optimal timing and duration of RN training Techniques for proper limb positioning Standing training methods Position transfer training exercises Joint mobility training procedures
➢ Traditional Chinese Medicine nursing	Hot compress application Acupressure techniques

## 4 Discussion

### 4.1 Principal findings

This study presents the first PVG for RN of stroke-related limb dysfunction in mainland China. Developed using a mixed-methods approach guided by PDKT (MC-PCG) and GIN’s PVG toolkit, this evidence-based guideline is available in both online and print formats. Unlike traditional health education, it offers evidence-based recommendations and decision support for patients (34). Key stakeholders, including patients, caregivers, health communicators, and healthcare providers, were involved from early stages. The application of the ICF framework ensured that the guideline development better addressed the RN information needs of stroke patients with limb functional impairment. Continuous stakeholder collaboration throughout the guideline development process is expected to enhance its adoption and usability in home rehabilitation for stroke patients with limb functional impairment.

### 4.2 Comparison with prior work

This study adopts an innovative method in the development process of Patient Version Guideline (PVG), which is different from the existing international model. We do not fully follow the guidance of the PVG reference book of the International Guidelines Network (GIN), but are patient oriented, based on a comprehensive assessment and identification of patient needs, a comprehensive search of multiple high-quality clinical guidelines to extract the best evidence to meet the needs of patients. Subsequently, we adopted a scientifically rigorous approach to integrate, detailing and contextualizing recommendations, and finally formed a patient-centered PVG. This approach takes into account the use of a single clinical practice guideline as the basis for PVG and may be limited in meeting patient needs. This view is consistent with the findings of Lijiao et al. (46).

Reasonable and standardized determination of the priority health problems of PVG is a necessary condition for the construction of PVG, which determines the scope of evidence retrieval and whether PVG can scientifically answer the health problems of patients. However, there is currently no unified methodological standard to guide the identification of priority PVG issues, and there is commonly a lack of focus on priority health issues in existing published PVG (47). In this study, a mixed research method was adopted to determine the health problems covered by PVG in the RN of stroke limb dysfunction. Based on the ICF framework, the study was organized on the basis of literature research and qualitative interviews, and then the demand level of RN was investigated through questionnaires, which avoided overly broad or specific limitations of the scope of problems to a certain extent.

In terms of the transformation of demand questions, we innovatively classified patient demand questions first, and then find clinical questions in relevant studies and guidelines according to the types of problems, so that the focus of demand questions is clear and not too broad. Studies have shown that the importance assessment of the overall problem can meet the need for the importance assessment of the guidelines (48). Therefore, the score on the importance of health problems related to RN for stroke limb dysfunction in this study can be used as the basis for evaluating the importance of PVG health problems, which is consistent with the research results of Xing (49).

Patient guidelines aim to facilitate shared decision-making by translating complex medical information into understandable language. However, the process of converting specialized RN knowledge into patient-friendly content remains challenging, with few studies detailing specific methods (50). Guided by the GIN Reference Book and the Patient Educational Materials Evaluation Tool (PEMAT) (35), this study adopted a systematic approach to content translation. PEMAT, a widely used and reliable tool for assessing patient education materials (35), provided both evaluation criteria and conversion principles for our Patient Version Guide (PVG). This methodology enhances the readability and practicality of the guide while offering a replicable approach for future research.

## 5 Limitation

This study acknowledges several methodological limitations. Primarily, participant recruitment was confined to the Beijing area of China, potentially limiting the generalizability of findings to diverse regional contexts. Future research should include a more geographically heterogeneous sample to enhance external validity across varied socio-geographic landscapes. Furthermore, while the Patient Version Guide (PVG) developed in this study shows promise in enhancing comprehension and engagement in RN knowledge among stroke patients and caregivers, its practical efficacy requires empirical validation. The hypothesized outcomes, including improved patient adherence and optimized rehabilitation practices, necessitate rigorous evaluation through longitudinal clinical studies. These should encompass analyses of implementation contexts and extended follow-up periods to ascertain the PVG’s effectiveness and guide its refinement. Systematic assessment of the PVG’s impact in diverse clinical settings is crucial to substantiate its utility and inform its broader dissemination. This approach will not only validate the tool’s effectiveness but also contribute to patient-centered rehabilitation care knowledge, potentially influencing future stroke rehabilitation guidelines.

## 6 Conclusion

Based on GIN handbooks and patient-oriented knowledge tools, this study developed a “Patient Guideline for RN of Post-Stroke limb dysfunction” through systematic evidence synthesis. This PVG serves as a practical resource for healthcare providers, patients, and caregivers, facilitating the implementation of evidence-based practices. However, the optimal methodology for developing such guides and their clinical value require continuous evaluation, given the complex factors involved in patients’ self-rehabilitation. Future research should focus on standardizing the development timing of patient guides, improving evidence evaluation methods, and assessing their application in real clinical settings. This study’s standardized approach provides a beneficial model for future research in this field.

## Data Availability

The original contributions presented in the study are included in the article/Supplementary material, further inquiries can be directed to the corresponding authors.

## Footnotes

^1^ http://www.guidelines-registry.org/guid/982
