# Supplementary material for: Empowering Stroke Survivors: developing a patient version of guidelines to facilitate patient rehabilitation nursing of stroke patients with limb dysfunction in China
Source: Front Public Health. 2025 Jan 7;12:1482771. doi: 10.3389/fpubh.2024.1482771 (PMC11747696; doi:10.3389/fpubh.2024.1482771)
Supplement: Supplementary file 1 [file Data_Sheet_1.docx]

**Appendix A Division of labor among patient version of guidelines ( PVG ) group members**

**Table A PVG group members and their responsibilities**

| Group category | Group members | Specific responsibilities |
| --- | --- | --- |
| Guide dvelopment group ( n=5 ) | Evidence-based nursing experts, clinical medicine experts, and nursing researchers | 1) Draft the PVG protocol and complete PVG registration.  2) Collect, select, and identify priority rehabilitation nursing issues.  3) Organize expert and patient/public consultations during the guideline development process and assist with external review. |
| Expert consensus group (n=5) | Methodology experts, evidence-based nursing experts, clinical nursing experts, nursing education experts, clinical medicine experts, hospital administrators, nursing researchers | 1) Form the PVG working group.  2) Review and approve the PVG development plan.  3) Define the target population, topics, and scope of the PVG.  4) Guide and oversee the methods and processes of PVG development.  5) Guide and supervise the evidence retrieval, evaluation, extraction, and the process and results of translating and refining recommendations.  6) Evaluate and reach consensus on the recommendations.  7) Review and revise all external review comments on the PVG.  8) Approve the release of the PVG. |
| Evidence evaluation and translation group (n=4) | Nursing researchers with a background in evidence-based medicine and training in relevant evidence-based methodology | 1) Complete evidence retrieval, evaluation, and extraction.  2) Complete the refinement and translation of evidence-based recommendations.  3) Assist the expert consensus group in addressing issues encountered during the PVG development process. |
| Patient and public group (n=3) | 2 patient representatives (patients clinically diagnosed with stroke and having limb dysfunction) and 1 science editor | 1) Participate in the collection of clinical questions.  2) Assess the translatability of evidence-based recommendations and the readability after detailed evidence content  or translate the detailed evidence content into an understandable format. |
| External review group (n=5) | Peer experts not involved in the development of this PVG (comprising 2 evidence-based methodology experts, 3 clinical experts in neurology, 2 patient representatives, and 1 science editor) | Review the draft guideline and provide feedback and suggestions for revisions;Patients and the science editor primarily evaluate the readability of the prototype PVG . |

**Appendix B Search strategy and inclusion criteria**

**1.Search strategy for evidence summary**

**Table B-1 Search strategy for evidence summary**

| **Category** | **Content** | |
| --- | --- | --- |
| **Search databases and websites** | **Major guideline websites** | （SIGN）Scottish Intercollegiate Guidelines Network；（NICE）National Institute For Health And Clinical Excellence；（NGC）National Guideline Clearinghouse；（NZGG）New Zealand Guidelines Group；（RNAO）Registered Nurses'Association Of Ontario；（GIN）Guideline International Network；（[Who）World Health Organization](http://www.baidu.com/link?url=-0xDW-eB4n3o5vaj3l6_AH6Y0OkPBzl5GHJxn9fQDom)；Clinical Evidence；Medlive |
|  | **Stroke professional association websites** | American heart association, Royal college of physicians (UK), National health and family planning commission stroke prevention and control project committee (China), European stroke organization, heart and stroke foundation of Canada, American stroke association. |
|  | **Chinese and English databases** | English databases: CINAHL; PubMed; Embase; Chinese databases: China National Knowledge Infrastructure（CNKI）； Wanfang Data（Wan Fang）；Chongqing VIP Information Co., Ltd.（VIP） ; Chinese Biology Medicine （CBM）( These databases, though less known internationally, are crucial for comprehensive access to Chinese academic research ） |
| **Search term scope and search terms** | Given that common symptoms of limb dysfunction due to stroke include hemiplegia, spasticity, and shoulder pain, and that traditional Chinese medicine also refers to limb dysfunction as "hemiplegia," these terms are included in the search terms. However, "sensory impairment" is not included in the search terms because its triggers are numerous and complex, and confounding factors cannot be completely eliminated. | |
| **Search terms** | Chinese search terms | （“卒中” OR “中风” OR “脑梗死” OR “脑梗塞” OR 脑栓塞 OR “脑血管意外” OR “脑出血” OR “脑血管疾病”）AND（“肢体功能障碍”OR “躯体功能障碍” OR“运动功能障碍” OR “半身不遂” OR 偏瘫 OR “痉挛” OR “肩疼”）AND（“指南” OR “证据”） |
|  | English search terms | （"stroke" OR "cerebrovascular disorders" OR "Stroke" OR "Cerebrovascular*" OR "cerebrovascular*" OR "apoplexy" OR "cerebral infarction"）AND（"Limb dysfunction" OR " hemiplegia" OR "paresis"）AND（"practice guideline" OR "CPG" OR "evidence"） |
|  | Search period | The search period for all websites and databases is from December 2016 to December 2021. |
|  | Example of search strategy in PubMed | #1 ((((((stroke[MeSH]) OR ("cerebrovascular disorders"[MeSH])) OR (stroke[Title/Abstract])) OR ("cerebrovascular disorders"[Title/Abstract])) OR (cerebrovascular*[Title/Abstract])) OR (brain vascular Accident[Title/Abstract])) OR ("cerebral infarction"[Title/Abstract]) OR ("apoplexy"[Title/Abstract])  #2 ((((((hemiplegia[MeSH]) OR (paresis[MeSH])) OR (spasm[MeSH])) OR (spasm[Title/Abstract])) OR (hemiplegia[Title/Abstract])) OR (paresis[Title/Abstract])) OR ("limb dysfunction"[Title/Abstract])  #3 (("practice guideline"[Title/Abstract]) OR (CPG[Title/Abstract])) OR (evidence[Title/Abstract])  #4 #1 AND 2 AND 3 |

1. **Inclusion and exclusion criteria for evidence**

**Table B-2 Inclusion and exclusion criteria for evidence**

| **Category** | **Content** |
| --- | --- |
| Inclusion criteria | Based on the Joanna Briggs Institute (JBI) PIPOST model for evidence-based healthcare and combined with previous research identifying 15 rehabilitation nursing issues, the final inclusion criteria are as follows:  ①Population (P): Patients clinically diagnosed with stroke and accompanied by limb dysfunction.  ②Intervention (I): Functional assessment, rehabilitation timing, functional exercise (exercise duration, positioning, standing training, position transfer), traditional Chinese medicine nursing, psychological nursing, and knowledge education.  ③Professional (P): The implementers of the evidence application are patients and their caregivers or clinical nursing staff.  ④Outcome (O): Limb function level; self-management level; prevention of complications (deep vein thrombosis, shoulder pain, falls, spasticity, and skin damage).  ⑤ Setting (S): Wards or home settings.  ⑥Type of Evidence (T): Guidelines, evidence summaries. |
| Exclusion criteria | ①Duplicate entries or directly translated foreign versions of guidelines or evidence summaries.② Conference abstracts, drafts, interpretations, etc., related to guideline development.③Studies on the implementation and application of evidence.④Evidence lists without methodological descriptions.⑤Evidence series for which the full text cannot be obtained.⑥ Non-Chinese or non-English evidence.⑦Evidence without levels of evidence or strength of recommendations. |

**Appendix C Principles and methods**

**1. Principles for extracting and excluding recommendations**

Table C-1 Principles for extracting and excluding recommendations

| Category | Content |
| --- | --- |
| Principles for extracting recommendations | 1) The recommendation is related to the 15 priority rehabilitation nursing issues.2) The recommendation is accurately described and the content is clear.3) It has clinical significance.4) It is a recommendation that patients can implement or easily understand.5) It is consistent with the clinical scenario in China.6) It aligns with patient values and wishes. |
| Principles for excluding recommendations | 1) Recommendations without an evidence level or strength of recommendation.2) Recommendations with very low evidence levels. |

**2. Principles for integrating recommendations**

Table C-2 Principles for integrating recommendations

| **Category** | **Content** |
| --- | --- |
| Consistency of recommendations | Select the most comprehensive recommendation that is concise and clear, with no substantial differences from the content of other recommendations. |
| Complementarity of recommendations | Combine related recommendations into a single, complete recommendation based on the logical relationships between their contents. |
| Conflict in recommendations | Trace the original literature of the recommendation, note the reasons for the conflict, and decide whether to retain or delete it after consulting with experts. |
| Independence of recommendations | Retain the original content of the recommendation. |

**3.Detailed evidence content**

Table C-3 Detailed evidence content

| **Category** | **Content** |
| --- | --- |
| **Method of detailing recommendations** | Detailed evidence content summary recommendations. When the recommendations lack sufficient operability or do not meet the patients' knowledge needs, trace back to the original guidelines or further trace the references of the evidence. If relevant references are lacking, supplement the search with original studies that meet the criteria. Since this patient guideline is intended for patients in China, prioritize the search for domestic original research evidence to reflect Chinese characteristics. For supplemental literature, two researchers will use the Cochrane handbook tool for assessing the quality of randomized controlled trials to evaluate and select higher quality literature for extraction.  The refinement process follows specific principles and is guided methodologically by evidence-based nursing experts. 2 nursing researchers are responsible for evidence tracing, supplemental searches, quality evaluation, and drafting and revising the refined content. Clinical experts will assess the reasonableness of the chapter settings, clinical significance, and accuracy of the refined content, providing judgments and suggestions. Patient and public representatives will primarily assess the adaptability of the refined content, offering their judgments and suggestions. |
| **Principles[12]** | Reasonable chapter settings:①The content is appropriately detailed and clear in its approach.② The content is rigorous, complete, and logically coherent.  Appropriateness:①The refined evidence content addresses the disease knowledge needed by patients or provides operational knowledge that patients can apply.② It is consistent with the context in China.  Clinical significance:①The refined evidence content can guide and improve limb rehabilitation, nursing, and self-management for patients with stroke-related limb dysfunction.②The refined evidence content will not lead to adverse experiences for patients with post-stroke limb dysfunction.  Accuracy:①The refined content remains faithful to the original guideline recommendations.②The refined evidence content only enhances the readability of the recommendations without altering their meaning. |

1. **Contextualizing the detailed evidence content into an understandable format**

Table C-4 Translate the detailed evidence content into an understandable format

| **Principles [35]** | **Content** |
| --- | --- |
| **Understandability** | 1) The translated content is clear and purpose-driven.2) The translated content uses simple and easy-to-understand language.3) Relevant technical terms are explained in layman's terms to make them familiar to patients.4) Content is expressed in the first person whenever possible.5) Numerical information is translated to be clear and easy to understand, using frequencies rather than probabilities (e.g., "Out of 10,000 people taking this medication, no more than 3 will have a heart attack" is easier for patients to understand than "The risk of a heart attack from taking this medication is 0.029%").6) The length of the translated content is moderate, with layered or segmented descriptions.7) The translated content is presented in a logical sequence.8) Visual cues or aids (such as arrows, large fonts, bold text, etc.) are used in the translated content.9) Images or charts used should have simple and clear titles. |
| **Feasibility** | 1) The translated content clearly describes the nursing measures that patients can take, primarily targeting patients (or caregivers).2) The translated rehabilitation/nursing measures should be broken down into actionable, clear steps.3) The translated content clearly explains how to use images, tables, charts, etc., to take action. |

**Reference：**

[12]G-I-N PUBLIC. G-I-N PUBLIC Toolkit: Patient and Public Involvement in Guidelines, https://g-i-n.net/wp-content/uploads/2021/04/GIN-TOOLKIT-COMBINED-FINAL-2015.pdf;2021[accessed 17 July 2024].

[35]Shoemaker SJ, Wolf MS, Brach C. Development of the Patient Education Materials Assessment Tool (PEMAT): a new measure of understandability and actionability for print and audiovisual patient information. Patient Educ Couns. 2014;96(3):395-403. https://doi.org/10.1016/j.pec.2014.05.027

**Appendix D Checklist of rehabilitation nursing issues for limb dysfunction in stroke**

**Table D Checklist of rehabilitation nursing issues for limb dysfunction in stroke**

| **Primary topic** | **Secondary topic** | **Rehabilitation nursing issue** | **Source** |
| --- | --- | --- | --- |
| Knowledge information | Disease knowledge | Can the use of appropriate assessment tools for functional evaluation improve my limb function/self-management level/quality of life? | literature/interview |
|  |  | Can understanding stroke prevention knowledge improve my limb function/self-management level/quality of life? | literature |
|  |  | What rehabilitation care can effectively prevent the occurrence of deep vein thrombosis? | interview |
|  |  | What rehabilitation care can effectively prevent the occurrence of falls? | interview |
|  |  | What rehabilitation care can effectively prevent spasms/relieve symptoms? | interview |
|  |  | What rehabilitation care can effectively prevent shoulder pain/relieve symptoms? | literature/interview |
|  |  | What nursing measures can effectively prevent skin breakdown? | literature/interview |
|  |  | Can understanding the harms of stroke-related limb dysfunction help improve my limb function/self-management level? | literature/interview |
|  |  | Can understanding the knowledge of rehabilitation equipment help improve my limb function/self-management level/quality of life? | literature/interview |
|  |  | Can early rehabilitation nursing help improve my limb function/self-management level/quality of life? | interview |
|  | Knowledge information channels | Can direct communication and health education from medical staff help improve my limb function/self-management level/quality of life? | literature |
|  |  | Can printed health education materials help improve my limb function/self-management level/quality of life? | literature |
|  |  | Can peer education help improve my limb function/self-management level/quality of life? | literature |
| Activity participation | Family life | Can daily activity exercises improve my limb function/self-management level/quality of life? | literature |
|  |  | Can home environment modifications improve my limb function/self-management level/quality of life? | literature/interview |
|  | Interpersonal communication and relationships | What nursing measures can improve my level of social participation? | literature |
| Limb function recovery | Rehabilitation training | Will different training times affect the rehabilitation of my limb function? | literature/interview |
|  |  | Can proper limb positioning help improve my limb function/self-management level/quality of life? | literature/interview |
|  |  | Can standing training help improve my limb function/self-management level/quality of life? | interview |
|  |  | Can position transfers help improve my limb function/self-management level/quality of life? | literature/interview |
|  | Traditional Chinese medicine nursing | What traditional Chinese medicine nursing methods can help improve my limb function/self-management level/quality of life? | literature/interview |
| Social environment | Family member support | Can family-assisted rehabilitation training help improve my limb function/self-management level/quality of life? | literature |
|  |  | Can family assistance with daily activities help improve my limb function/self-management level/quality of life? | literature |
|  |  | Can family education and psychological support help improve my limb function/self-management level/quality of life? | literature |
|  | Medical system support | Can continuous rehabilitation nursing help improve my limb function/self-management level/quality of life? | literature |
| Psychological and emotional well-being | Psychological nursing | Can psychological nursing help improve my limb function/self-management level/quality of life? | literature/interview |

**Appendix E Evidence Integration Results**

**(1) Quality assessment results of the included guidelines and ICC consistency test**

4 B-level evidence-based guidelines were ultimately obtained and included after internal discussion.

Table E-1 Quality assessment results of the included guidelines and ICC consistency test

| Guideline | Standardized scores for each domain | | | | | | Number of domains with ≥60% | Number of domains with <30% | Intraclass correlation coefficient | Level of evidence |
| --- | --- | --- | --- | --- | --- | --- | --- | --- | --- | --- |
|  | Scope and purpose（%） | Stakeholder involvement（%） | Rigor of development（%） | Clarity of presentation（%） | Applicability（%） | Editorial independence（%） |  |  |  |  |
| 1^[3]^ | 81.48 | 40.74 | 54.86 | 87.04 | 56.94 | 61.11 | 3 | 0 | 0.796 | B |
| 2^[4]^ | 77.78 | 59.26 | 70.83 | 87.04 | 36.11 | 80.56 | 4 | 0 | 0.890 | B |
| 3^[5]^ | 79.63 | 25.22 | 28.47 | 77.78 | 44.44 | 77.78 | 3 | 2 | 0.823 | B |
| 4^[6]^ | 81.48 | 47.37 | 47.22 | 66.67 | 54.17 | 88.89 | 3 | 0 | 0.920 | B |

**(2) Evidence integration assessment results**

This study ultimately included 4 evidence summaries, all of which were incorporated after internal discussion.

Table E-2 Evidence summary assessment results

| Assessment items（case worksheet） | 5^[7]^ | 6^[8]^ | 7^[9]^ | 8^[10]^ |
| --- | --- | --- | --- | --- |
| Domain 1: Summary topic | | | | |
| 1.Is the summary specific in scope and application? | Yes | Yes | Yes | Yes |
| Domain 2: Summary methods | | | | |
| 2. Is the authorship of the summary transparent? | Yes | Yes | Yes | Yes |
| 3.Are the reviewer(s)/editor(s) of the summary transparent? | Yes | Yes | No | No |
| 4.Are the search methods transparent and comprehensive? | Not completely | Not completely | Not completely | Not completely |
| 5.Is the evidence graded and is the system transparent and translatable | Yes | Yes | Yes | Yes |
| Domain 3：Summary content | | | | |
| 6.Are the recommendations clear? | Yes | Yes | Yes | Yes |
| 7.Are the recommendations appropriately cited? | Yes | Yes | Yes | Yes |
| 8.Are the recommendations current? | Yes | Yes | Yes | Yes |
| 9.Is the summary free of possible bias? | No | No | No | No |
| Domain：Summary application |  |  |  |  |
| 10.Can this summary be applied to your patient(s)? | Yes | Yes | Yes | Yes |
| Assessment Results： | Include | Include | Include | Include |
| Note: Inclusion criteria: No more than three questions in domain 2 and domain 3 are answered "No," and the proportion of "Not entirely" options is ≤30%.  Exclusion criteria: More than 5 questions in any domain are answered "No," or the proportion of "Not entirely" options is ≥60%. | | | | |

**（3）Comprehensive results of recommendations**

Table E-3 Best evidence summary for rehabilitation nursing of limb dysfunction in stroke patients

| Theme | Recommendations (strength of recommendation, source of evidence) |
| --- | --- |
| 1.  Function assessment | 1.It is recommended to use standardized and effective assessment tools to evaluate stroke-related disabilities and functional status, determine the appropriate level of care, and inform patients and their families/caregivers of the expected outcomes to promote limb rehabilitation. These functional assessments can include the following tools：   - The 6-level muscle strength assessment scale can be used to evaluate muscle strength, facilitating the selection of appropriate muscle strength training methods. - The barthel index can be used to assess the patient's ability to perform daily activities, determine the level of care needed, develop individualized care plans, and provide safety warnings about adverse events to healthcare providers and family members. - The fim (functional independence measure) scale can be used to assess the patient's ability to perform daily activities, facilitating the development of individualized care plans.   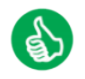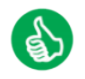  [(②③⑦)] |
| 2.  Symptom prevention and care | 2.1Spasticity：   - 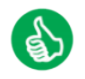For stroke patients, the management of spasticity should begin in the early stages of the condition. It is recommended that stroke patients maintain proper limb positioning, which can effectively reduce limb spasticity.   [(①)]   - 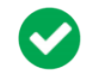For stroke patients who develop spasticity, it is recommended to use passive stretching and traditional Chinese massage therapy to alleviate the spasticity.   [(①)] |
|  | 2.2 Shoulder pain：   - Avoid excessive shoulder flexion and abduction movements, as well as pulley-like exercises with hands raised overhead, as these can lead to uncontrolled shoulder abduction and result in shoulder pain.   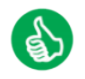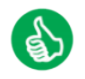[(①②）]   - In the early stages after a stroke, perform active and passive joint mobility and 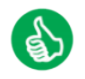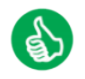stretching exercises. Encourage the patient to perform maximum external rotation exercises of the hemiplegic arm for 30 minutes.   [(①②)]   - For patients with shoulder subluxation, consider using positional support, supportive devices, and slings, and avoid high pulley exercises.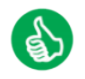   [(②⑥)]  2.3 Deep vein thrombosis (DVT)：   - 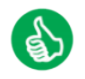For stroke patients, early positional changes and joint mobilization can help prevent the occurrence of DVT.   [(⑦)]   - 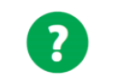There is no evidence to support that the use of compression stockings can treat or prevent the occurrence of DVT and pulmonary embolism.   [(①④)]  2.4 Skin damage：   - For stroke patients, maintaining adequate nutrition and hydration, positioning the body properly, and regularly turning can effectively reduce the pressure on the same bony prominences and lower the risk of skin damage.   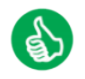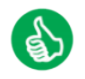[(①)]   - For stroke patients who are bedridden, it is recommended to use air mattresses and foam pads to reduce or avoid skin friction, decrease skin pressure, provide adequate support, and prevent localized excessive moisture. Circular air rings should be avoided. Maintain good skin hygiene and use specialized mattresses, wheelchair cushions, and seats until function is restored.   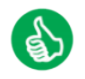[(①④)]  2.5 Falls：   - During hospitalization, it is recommended that each stroke patient undergo a fall risk assessment and receive an individualized fall prevention plan. For stroke patients transitioning to community living, those with poor balance function, low confidence, fear of falling, or at risk of falling should undergo balance training. Practices such as Tai Chi may be a reasonable method for preventing falls.   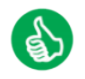[(①④)] |
| 3.  Rehabilitation training | 3.1 Training time/timing：   - Rehabilitation training intensity should be individualized, fully considering the patient's physical strength, endurance, and cardiopulmonary function. When conditions permit, starting with at least 45 minutes of rehabilitation training per day can improve the patient's function. It is beneficial to appropriately increase training intensity, and if necessary, conduct the training under supervision.   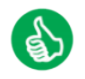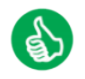[(①)]   - For patients with mild to moderate conditions, bedside rehabilitation, early mobilization, and proper limb positioning can be initiated within 24 hours (or within 3 days) of onset. However, intensive mobility training (such as frequent getting out of bed) should be avoided too early.   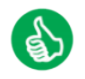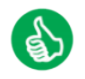[(⑥)]  3.2 Training intensity：   - When safe and considering the patient's physical strength, endurance, and cardiopulmonary function, provide any opportunity to increase training frequency. However, it should be done gradually, as appropriately increasing training intensity is effective.   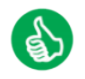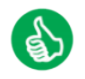[(⑥⑦)]  3.3 Training methods：  3.3.1 Positioning：   - For stroke patients with hemiplegia, positioning should follow proper limb alignment. This should be initiated 48 hours after the patient's vital signs stabilize and neurological symptoms cease to progress. This approach can reduce the incidence of limb dysfunction in stroke patients with hemiplegia. It is recommended to change positions every 1-2 hours.   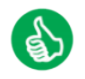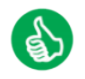[(①②③)]   - Supine position: Place a thin pillow under the head; support the affected scapula and upper limb with a long pillow, with the upper arm rotated outward and abducted 20°-40°, and both the elbow and wrist extended; fingers extended with palms facing up; place pillows under the hips, buttocks, and outer thighs; slightly elevate under the knees; keep the toes pointing upward.   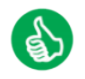[(⑤)]   - Lying on the unaffected side: (Unaffected side down, affected side up) Place a pillow under the head; extend the affected upper limb and position it on a pillow, with the affected scapula protracted 90°-100° and the forearm pronated; fingers extended with palms facing down; position the affected lower limb in slight flexion on a long pillow, ensuring the affected ankle does not invert and hangs over the edge of the pillow to prevent foot drop and inversion.   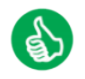[(⑤)] |
|  | - Wheelchair seating: The patient should keep their torso straight, leaning against the back of the chair, and sit as far back as possible on the wheelchair cushion, maintaining a slight forward lean. The affected upper limb should be placed on a soft pillow in front of the chest, either extended forward or flexed close to the body, avoiding excessive elbow flexion, with fingers naturally extended. Place a soft cushion on the outer side of the affected leg to correct external rotation. The hip, knee, and ankle joints should all be at 90°, with both feet perpendicular to the knees, flat on the floor, and toes pointing forward. The feet should be shoulder-width apart, avoiding external rotation of the toes, and keeping both toes symmetrical.   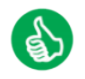[(⑤)]  3.3.2 Joint mobility：   - Patients who are bedridden due to stroke should continue with joint mobility exercises, but care must be taken to protect the affected limbs from mechanical injury.   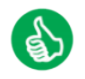[(①②⑦)]  3.3.3 Standing training：   - In the early stages of stroke with hemiplegia, and once the condition has stabilized, patients should get out of bed as soon as possible and actively engage in standing training to regain basic walking ability as early as possible.   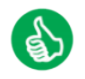[(①⑦)]  3.3.4 Position transfer：   - Patients who are bedridden due to stroke should begin progressive position transfer training with the help of caregivers as early as possible to prevent complications such as aspiration pneumonia, DVT, and pressure sores. However, safety precautions must be taken during the training process.   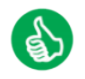[(①②⑦)] |
| 4.  Traditional Chinese medicine nursing | 4.1 Acupressure：   - For patients with hemiplegia after a stroke, acupressure can be used to promote blood circulation in the limbs and improve symptoms such as stiffness and numbness, provided conditions allow.   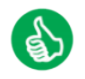[(⑧)]  4.2 Herbal hot compress：   - For patients with hemiplegia after a stroke, herbal fumigation and washing can be used to improve the degree of neurological deficits, thereby reducing pain and increasing muscle strength, provided conditions allow.   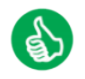[(⑧)]  4.3 Herbal fumigation and washing：   - For patients with hemiplegia after a stroke, herbal fumigation and washing can be used to warm the meridians, promote circulation, reduce swelling, relieve pain, and prevent deep vein thrombosis in the lower limbs, provided hospital resources, facilities, and personnel allow.   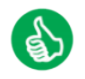[(⑧)] |
| 5.  Disease knowledge | 5.1 Enhance health education and nursing guidance for rehabilitation care, informing patients that undergoing rehabilitation treatment in the community after being discharged from the hospital can also be effective for stroke recovery.  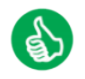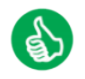[(①)]  5.2 Patients and their family members should be informed of the disease assessment results. Both the patient and caregivers should actively participate in health education (including disease knowledge, prevention, and the importance of rehabilitation), strengthen their awareness of participation, and timely acquire the latest disease knowledge. This will improve the overall quality of rehabilitation for stroke patients and promote better family and social support for the patient.  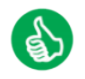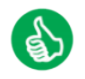[(②③⑦)] |

Notes: ①Refers to the "Chinese clinical management guidelines for cerebrovascular diseases";② Refers to the "Chinese guidelines for early rehabilitation treatment of stroke";③Refers to the "Chinese stroke nursing guidance standards";④Refers to the "Guidelines for adult stroke rehabilitation and recovery";⑤Refers to the "Best evidence summary for proper limb positioning management in patients with hemiplegia due to cerebral infarction";⑥Refers to the "Best evidence summary for early rehabilitation nursing in stroke patients with limb dysfunction";⑦Refers to the "Evidence summary for limb function exercises in stroke patients";⑧Refers to the "Evidence-based traditional Chinese medicine nursing plan for stroke".

**Reference：**（It is consistent with the reference sequence number in the main text of the study）

[16]Winstein CJ, Stein J, Arena R, Bates B, Cherney LR, Cramer SC, et al. Guidelines for Adult Stroke Rehabilitation and Recovery: A Guideline for Healthcare Professionals From the American Heart Association/American Stroke Association. Stroke. 2016;47(6):e98–e169. <https://doi.org/10.1161/STR.0000000000000098.>

[17]Liping L, Weiqi C, Hongyu Z, Wanying D, Shujuan L, Xiaochuan H, et al. Chinese Stroke Association guidelines for clinical management of cerebrovascular disorders: executive summary and 2019 update of clinical management of ischaemic cerebrovascular diseases. Stroke Vasc Neurol. (2020) 5:159–76. doi: 10.1136/svn-2020-000378

[40]Zhang T, Zhao J. Guidelines for early rehabilitation of stroke in China. Chinese Journal of Neurology. 2017;50(6):405-412. https://doi.org/10.3760/cma.j.issn.1006-7876.2017.06.002.

[41]Stroke Prevention and Control Engineering Committee of the National Health and Family Planning Commission. Chinese Stroke Nursing Guidelines 2021. http://www.nhc.gov.cn/yzygj/s3593/202108/50c4071a86df4bfd9666e9ac2aaac605.shtml.

[42]Li G, Chen H, Liu y, Song X, Jia L. Evidence Summary for Good Limb Position Management in Hemiplegic Patients with Cerebral Infarction. Journal of Nursing. 2019;26(24):22-27. https://doi.org/10.16460/j.issn1008-9969.2019.24.022.

[43]Chen H, Xie H, Li W, Luo Q, Ji Y. Evidence Summary for Early Rehabilitation Nursing of Stroke Patients with Limb Dysfunction. Military Nursing. 2020;37(06):6-10. https://doi.org/10.3969/j.issn.1008-9993.2020.06.002.

[44]Liu X, Zhu G, Cai Y, Yi M, Li L. Synthesis of the evidence regarding functional exercise for stroke survivors. Chinese Nursing Management. 2020;20(11):1689-1694. https://doi.org/10.3969/j.issn.1672-1756.2020.11.020.

[45]Li X. Evidence-based Optimization of TCM Nursing Programs for Stroke[D]. Beijing University of Chinese Medicine,2019.

**Appendix F Results of detailed evidence content**

Table F Results of detailed evidence content

| Theme | Chapter setup | Section setup | Corresponding recommendations |
| --- | --- | --- | --- |
| 1.Disease knowledge | 1.Understanding limb dysfunction in stroke | 1.1. Why is functional assessment important? | Recommendation 5.2 |
|  |  | 1.2. Understanding limb dysfunction |  |
|  |  | 1.3. How to conduct rehabilitation training |  |
|  |  | 1.4. The importance of rehabilitation nursing to me | Recommendation 5.1 |
| 2.Functional assessment | 2.Why is functional assessment important? | 2.1 Methods and significance of ADL assessment | Recommendation ：1.1 |
|  |  | 2.2 Significance of muscle strength assessment | Recommendation ：1.1 |
| 3.Rehabilitation training | 3.How to conduct rehabilitation training | 3.1 When to start rehabilitation training | Recommendation ：3.1 |
|  |  | 3.2 What are the types of rehabilitation training? | Recommendation ：3 |
|  |  | 3.3 Proper limb positioning training   - What is proper limb positioning - Benefits of proper limb positioning - How to perform proper limb positioning | Recommendation ：3.3.1 |
|  |  | 3.4 Joint mobility training   - What is joint mobility training - Benefits of joint mobility training - Methods of joint mobility training | Recommendation ：3.3.2 |
|  |  | 3.5 Standing training   - What is standing training - Benefits of standing training - Methods of standing training | Recommendation ：3.3.3 |
|  |  | 3.6 Position transfer training   - What is position transfer - Benefits of position transfer - How to perform position transfer | Recommendation ：3.3.4 |
|  |  | 3.7 What to pay attention to during rehabilitation training | Recommendation ：3.2 |
| 4.Traditional Chinese medicine nursing | 4.How to use traditional Chinese medicine nursing techniques to alleviate symptoms | 4.1 Acupressure | Recommendation ：4.1 |
|  |  | 4.2 Herbal hot compress | Recommendation ：4.2 |
| 5.Prevention and care of risks and complications | 5.What to do when complications arise? | 5.1 Be alert to fall accidents   - Dangers of falling - Prevention methods | Recommendation ：2.5 |
|  |  | 5.2 How to prevent deep vein thrombosis (DVT)   - Dangers of DVT - Prevention methods | Recommendation ：2.3 |
|  |  | 5.3 How to prevent skin damage   - Dangers of skin damage - Prevention methods - Care methods | Recommendation ：2.4 |
|  |  | 5.4 How to prevent spasms   - Dangers of spasms - Care methods | Recommendation ：2.1 |
|  |  | 5.5 How to prevent shoulder pain   - Dangers of shoulder pain - Prevention methods | Recommendation ：2.2 |

**Appendix G Prototype of PVG**


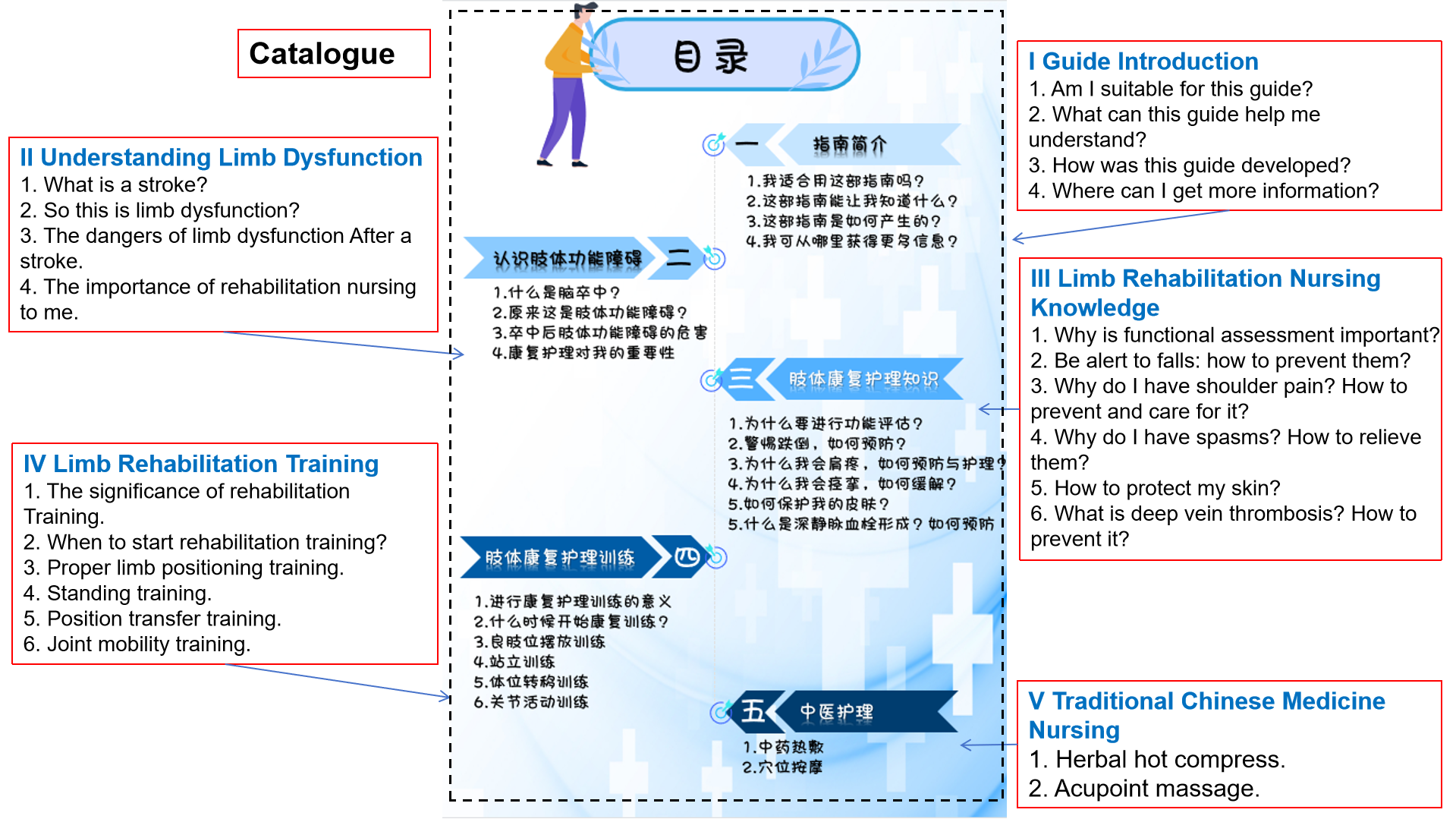


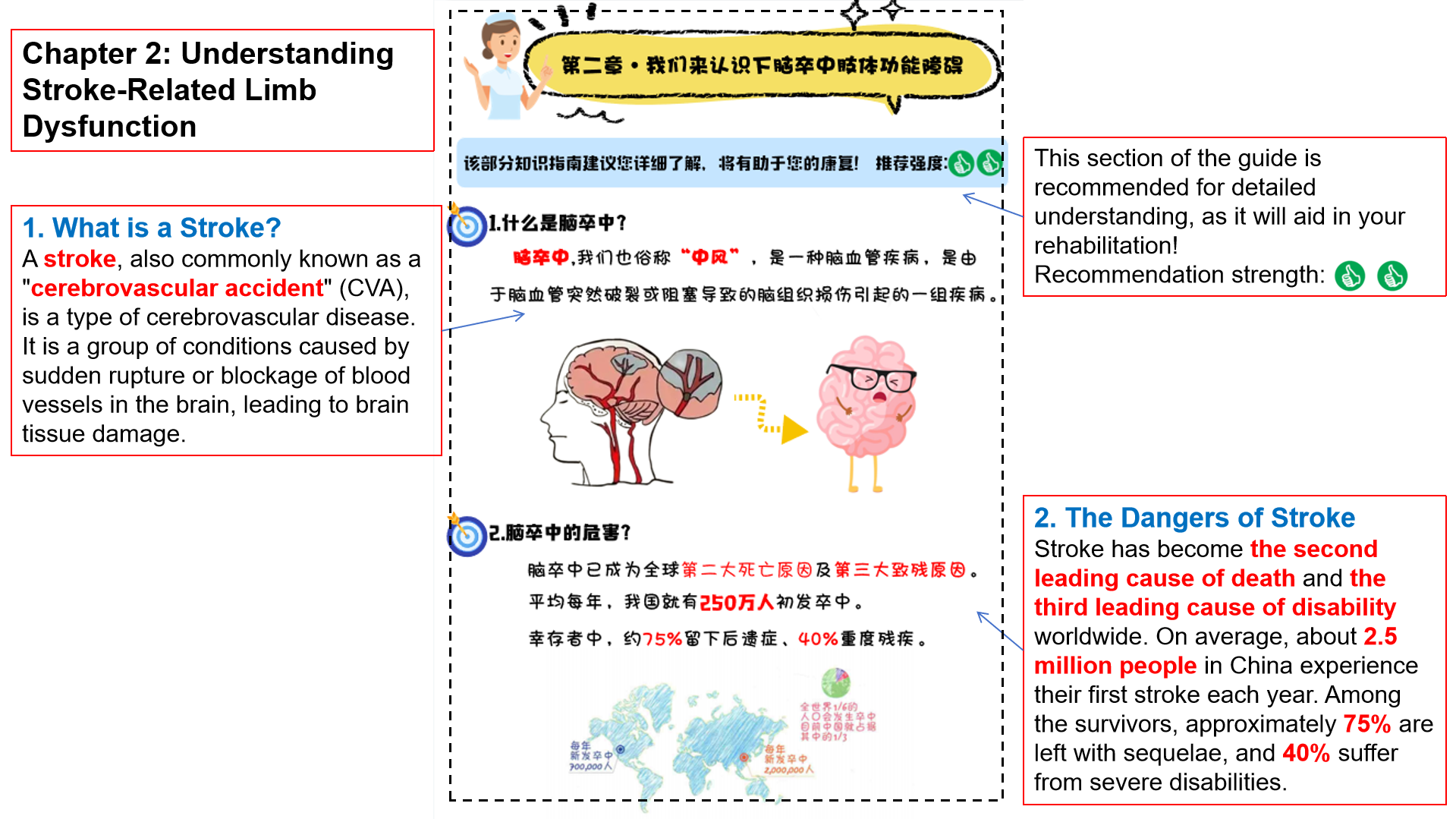


**Figure G PVG table of contents and selected chapters**

Notes:The text inside the dashed box is the PVG Chinese content, and the text inside the red box is the translated English content.

**Appendix H Experts' evaluation results on the scientific and feasibility aspects of PVG**

Table H Priority scoring table for rehabilitation nursing issues

| Number | Priority issues in rehabilitation nursing | Importance scores（‾x±s） |
| --- | --- | --- |
| 1 | Can understanding stroke prevention knowledge improve my limb function/self-management level/quality of life? | 5.00 |
| 2 | Does early rehabilitation nursing help improve my limb function/self-management level/quality of life? | 5.00 |
| 3 | Which rehabilitation nursing practices can effectively prevent falls? | 5.00 |
| 4 | Which nursing measures can effectively prevent skin damage? | 5.00 |
| 5 | Which rehabilitation nursing practices can effectively prevent/relieve spasm symptoms? | 4.40±0.55 |
| 6 | Does proper limb positioning help improve my limb function/self-management level/quality of life? | 4.60±0.55 |
| 7 | Does standing training help improve my limb function/self-management level/quality of life? | 4.20±0.45 |
| 8 | Does using appropriate assessment tools for limb function evaluation help improve my limb function/self-management level/quality of life? | 5.00 |
| 9 | Does position transfer help improve my limb function/self-management level/quality of life? | 4.20±0.45 |
| 10 | Does joint mobility training help improve my limb function/self-management level/quality of life? | 4.06±0.13 |
| 11 | Does the timing of training affect the rehabilitation of my limb function? | 4.00 |
| 12 | Which rehabilitation nursing practices can effectively prevent/relieve shoulder pain symptoms? | 4.00 |
| 13 | Does psychological nursing help improve my limb function/self-management level/quality of life? | 5.00 |
| 14 | Which traditional Chinese medicine nursing practices help improve my limb function/self-management level/quality of life? | 4.00 |
| 15 | Which rehabilitation nursing practices can effectively prevent deep vein thrombosis (DVT)? | 4.00 |

**Appendix I Patient guide for rehabilitation nursing of limb dysfunction after stroke, 2022 edition (first edition)**

**【PVG manual table of contents (part)】**


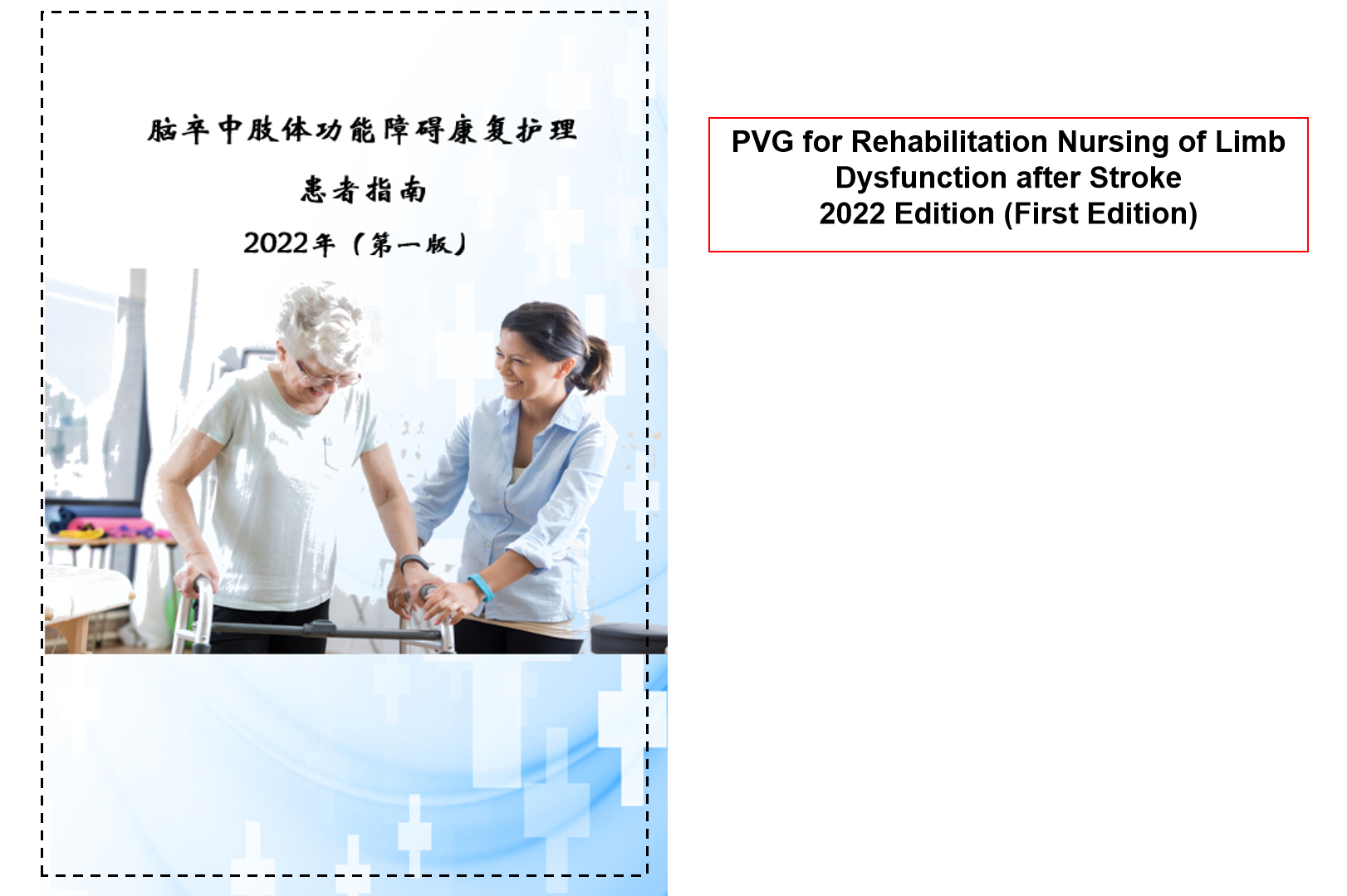


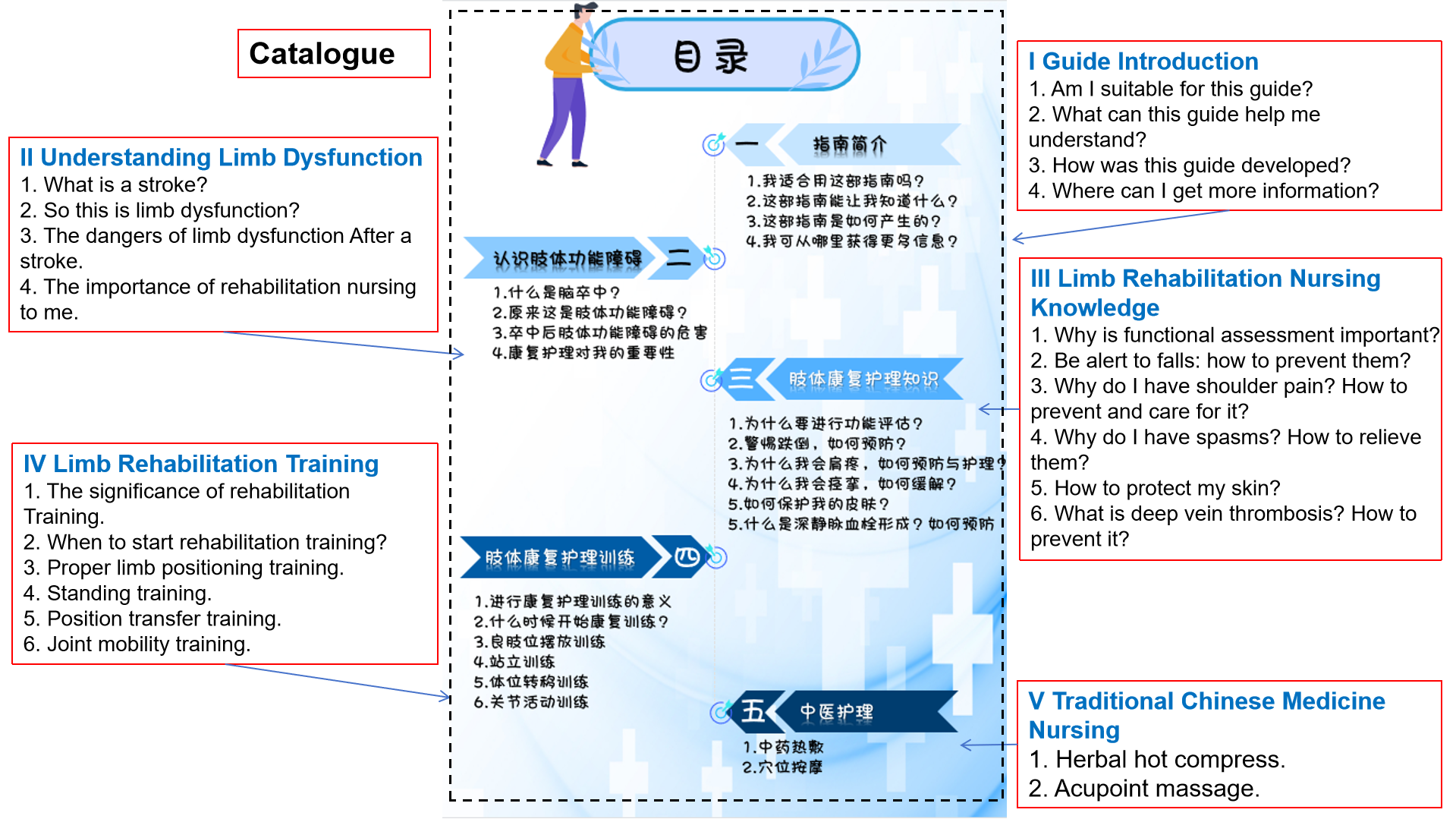


**Figure I-1 PVG cover and table of contents**


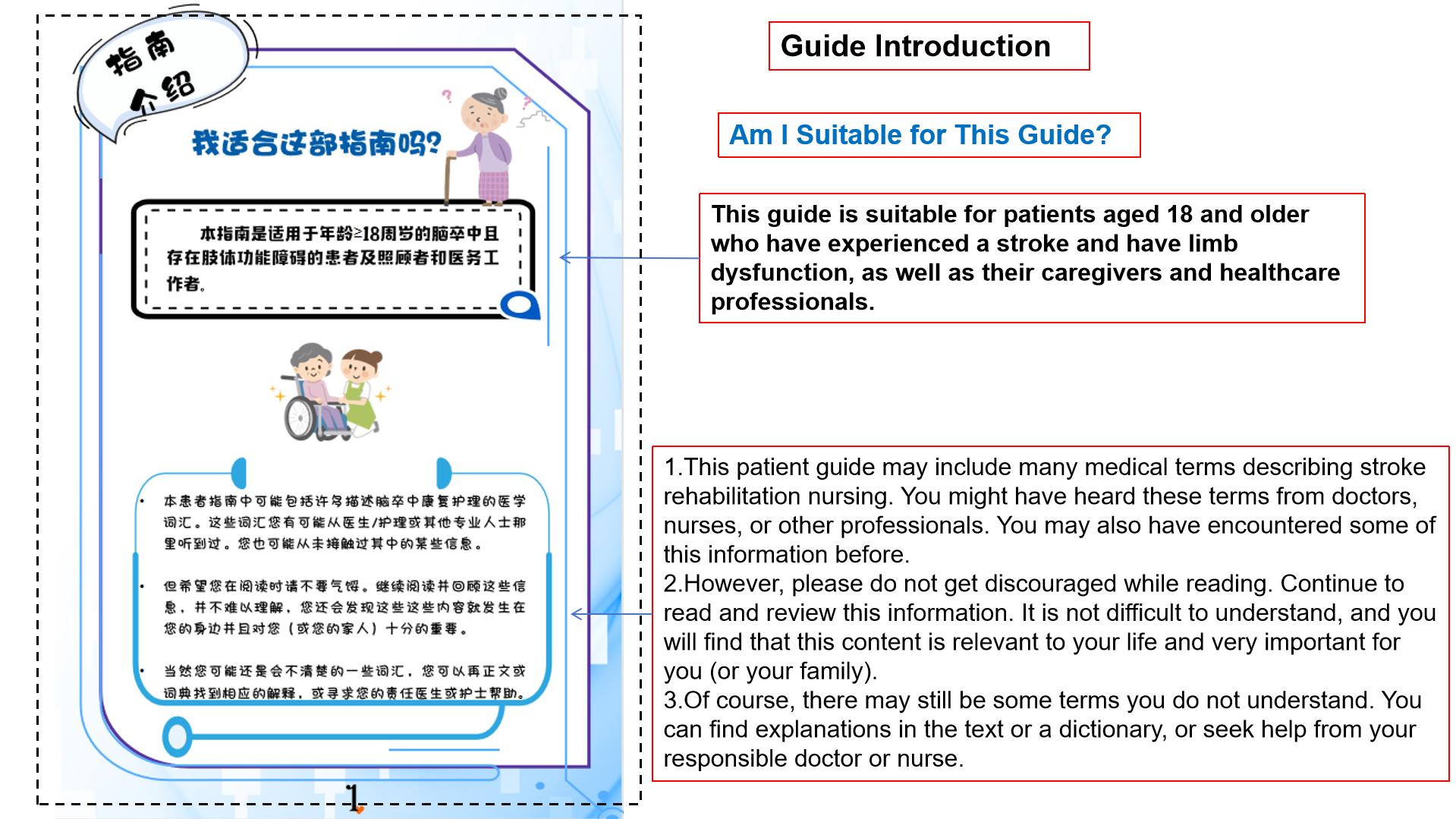


**Figure I-2 Introduction to PVG**


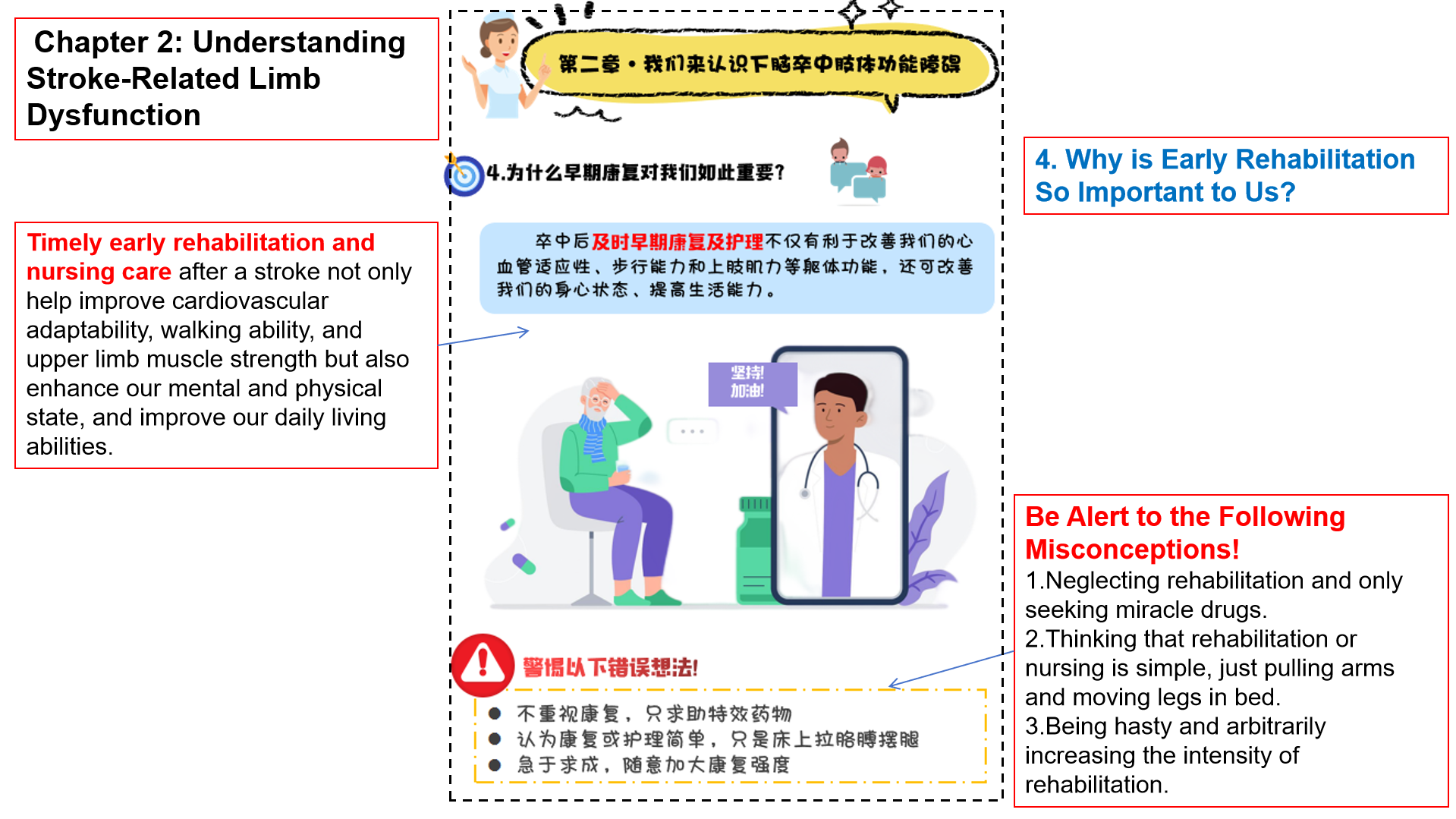


**Figure I-3 PVG content (part)**

**【Wechat popular science tweets of PVG】**


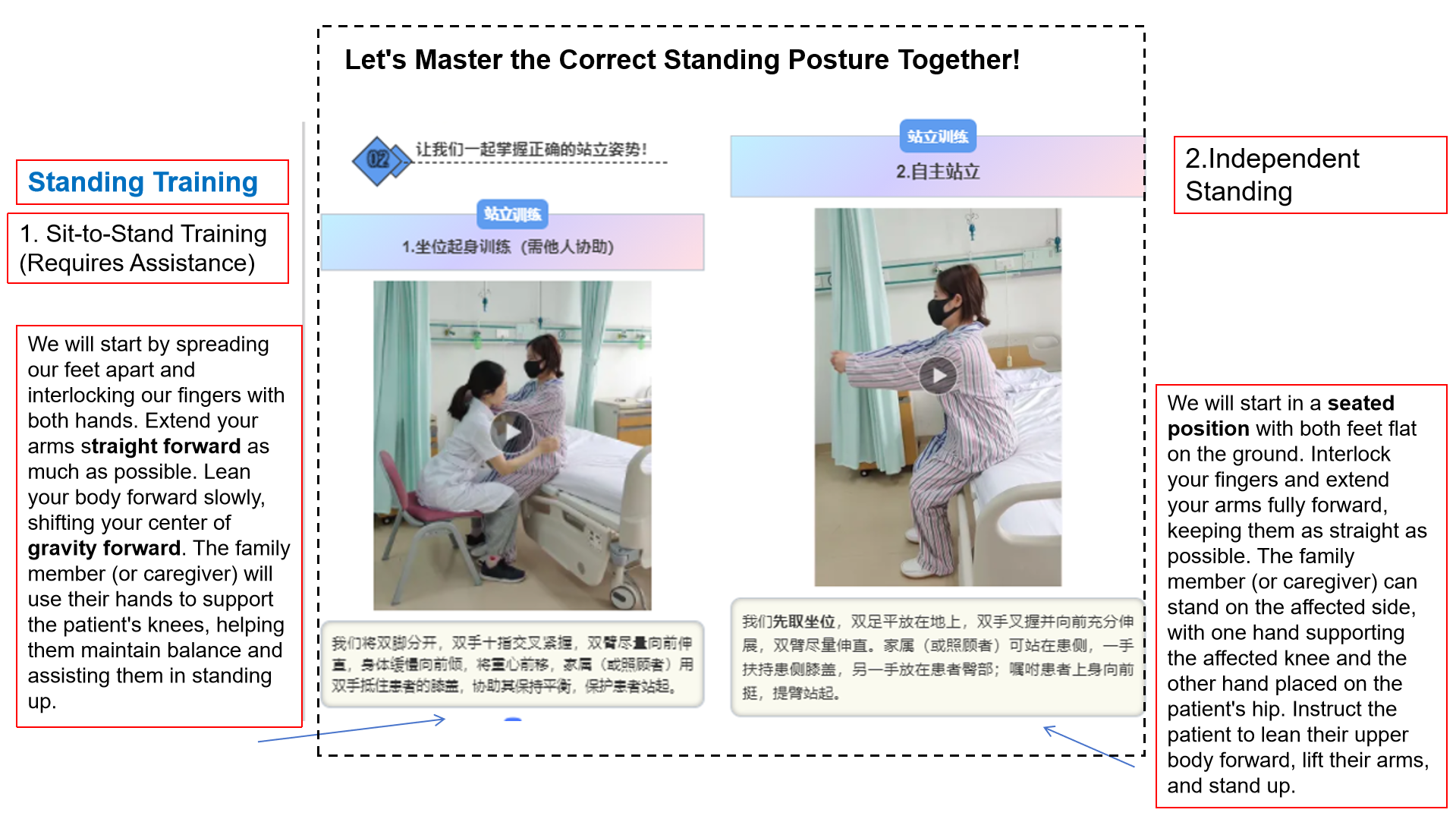


**Figure I-4 Rehabilitation nursing training video Tweets(part)**


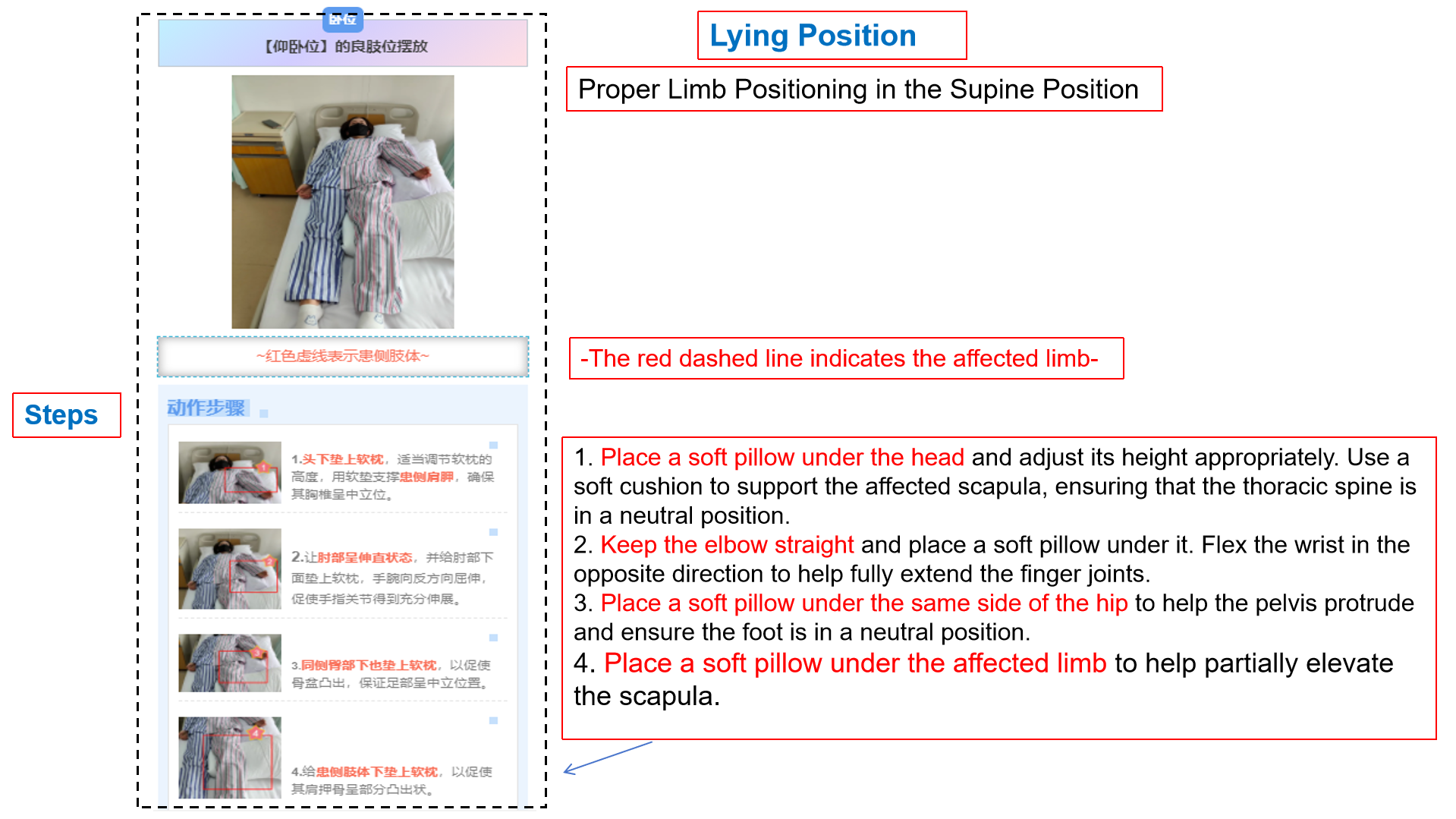


**Figure I-5 Illustration of rehabilitation nursing training steps (Part)**

Notes:The text inside the dashed box is the PVG Chinese content, and the text inside the red box is the translated English content.
